# Theta Burst Stimulation of the Cerebellum Modifies the TMS-Evoked N100 Potential, a Marker of GABA Inhibition

**DOI:** 10.1371/journal.pone.0141284

**Published:** 2015-11-03

**Authors:** Allanah Harrington, Graeme David Hammond-Tooke

**Affiliations:** 1 Department of Medicine, University of Otago, Dunedin, New Zealand; 2 Department of Neurology, Dunedin Hospital, Dunedin, New Zealand; University of Ottawa, CANADA

## Abstract

Theta burst stimulation (TBS) of the cerebellum, a potential therapy for neurological disease, can modulate corticospinal excitability via the dentato-thalamo-cortical pathway, but it is uncertain whether its effects are mediated via inhibitory or facilitatory networks. The aim of this study was to investigate the effects of 30Hz cerebellar TBS on the N100 waveform of the TMS-evoked potential (TEP), a marker of intracortical GABA_B_-mediated inhibition. 16 healthy participants (aged 18–30 years; 13 right handed and 3 left handed) received 30Hz intermittent TBS (iTBS), continuous TBS (cTBS) or sham stimulation over the right cerebellum, in three separate sessions. The first 8 participants received TBS at a stimulus intensity of 80% of active motor threshold (AMT), while the remainder received 90% of AMT. Motor evoked potentials (MEP) and TEP were recorded before and after each treatment, by stimulating the first dorsal interosseus area of the left motor cortex. Analysis of the 13 right handed participants showed that iTBS at 90% of AMT increased the N100 amplitude compared to sham and cTBS, without significantly altering MEP amplitude. cTBS at 80% of active motor threshold decreased the N100 amplitude and cTBS overall reduced resting MEP amplitude. The study demonstrates effects of 30Hz cerebellar TBS on inhibitory cortical networks that may be useful for treatment of neurological conditions associated with dysfunctional intracortical inhibition.

## Introduction

Repetitive transcranial magnetic stimulation (rTMS) is a potential therapy for neurological and psychiatric disorders, increasing or decreasing cortical excitability, depending on the stimulation frequency [1–4]. In general, high frequency stimulation of the primary motor cortex (>5Hz) increases corticospinal excitability, as demonstrated by increased motor evoked potential (MEP) amplitudes, and low frequency stimulation (1Hz or less) does the opposite. A range of neurological disorders, including epilepsy and dystonia, may involve dysfunctional intracortical inhibition, and may respond to treatments that modify it [5,6].

Theta burst stimulation (TBS), in which subthreshold high frequency pulses are applied in bursts at theta frequency, has been shown to have advantages over fixed frequency rTMS, requiring shorter duration of treatment to produce a longer-lasting effect [7]. Typically, continuous TBS (cTBS) applied to the motor cortex decreases, while intermittent TBS (iTBS) increases motor evoked potential (MEP) amplitude. TBS originally employed bursts of pulses at 50Hz, but similar effects may be obtained with 30Hz TBS, which permits higher stimulation intensity (SI) with certain stimulators [8,9].

A disadvantage of rTMS applied to the cerebral cortex is that the effects are mostly localised to the stimulated area. In some disorders, the abnormality may be more diffuse and focal stimulation less effective. The cerebellum has extensive connections with the cerebral cortex, through which cerebellar stimulation could modulate excitability in cortical areas beyond those traditionally associated with movement [10]. These connections have been demonstrated by tracer studies in primates [11] and in humans, using magnetic resonance tractography and functional magnetic resonance imaging [12,13].

**Fig 1:**
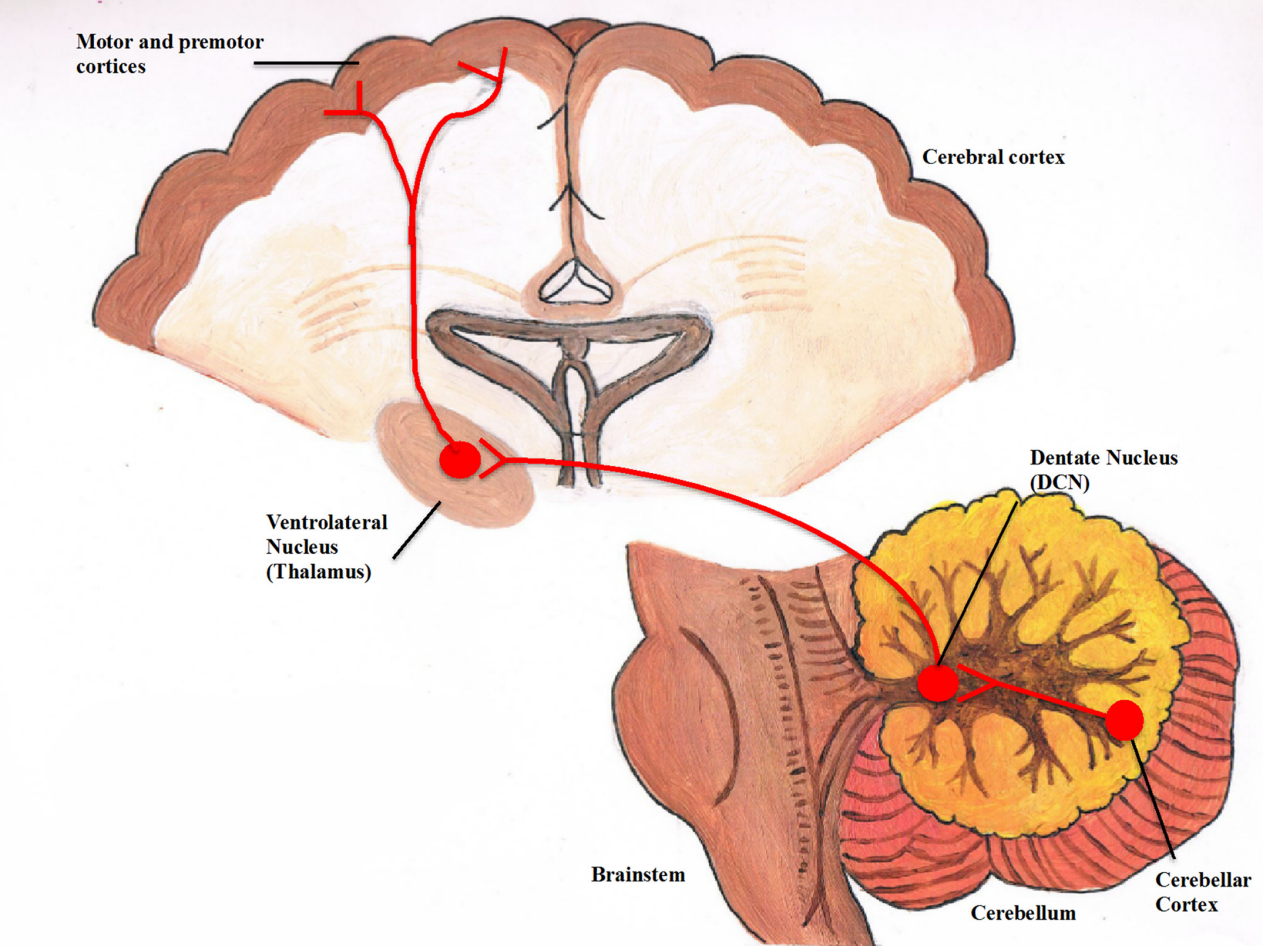
Diagramatic representation of the cerebello-dentato-thalamo-cortical pathway. The figure depicts the pathway from the cerebellum to the motor cortex, via the ventrolateral nucleus of the thalamus. The Purkinje cells in the cerebellar cortex have inhibitory synapses on the cells of the deep cerebellar nuclei, and are thought to modulate the tonic excitatory effects of the dentate-thalamo-cortical pathway on cortical excitability. (+) = excitatory synapses; (-) = inhibitory synapses. (Diagram by A. H.).

The connections between the cerebellum and the motor cortex can be studied by applying an electrical stimulus to the cerebellar cortex milliseconds (msec) before a test pulse to the contralateral primary motor cortex [14]. The amplitude of the MEP obtained by the test pulse is reduced when using an inter-stimulus interval of about 5–7 msec; a phenomenon known as cerebellocortical inhibition (CBI). It is thought that the dentato-thalamo-cortical pathway exerts a tonic excitatory effect on the motor cortex, and this is briefly inhibited by stimulating the cerebellar cortex, whose Purkinje cells have inhibitory synapses on the cells of the dentate nucleus (Fig 1) [15]. However the effects of single pulse stimulation of the cerebellar cortex are complex, with effects on both excitatory and inhibitory networks [16].

The reported effects of cerebellar rTMS on cortico-motor excitability have been variable. Two studies [17,18] suggested that 1Hz cerebellar rTMS increased MEP amplitude obtained from the contralateral motor cortex, although a third study [19] demonstrated no change. Thus, 1Hz rTMS, which usually reduces MEP amplitudes when applied directly to the motor cortex, increased them when applied to the contralateral cerebellum, at least in two studies. It could be that inhibition of Purkinje cells released the dentate-thalamo-cortical pathway from Purkinje cell inhibition, resulting in increased cortical excitability.

Koch and colleagues [20] examined the effects of cerebellar TBS on MEP amplitudes and paired pulse measures of intracortical inhibition and facilitation in the contralateral motor cortex. Following cTBS at a stimulus intensity (SI) of 80 or 90% of active motor threshold (AMT), there were decreased MEP amplitudes and short interval cortical inhibition (SICI) and increased long interval cortical inhibition (LICI). iTBS at 80% of AMT produced an increase in MEP amplitude and a decrease in intra-cortical facilitation (ICF) and LICI. Thus, cerebellar cTBS decreased corticospinal excitability as reflected in MEP amplitude, and cerebellar iTBS produced the opposite effect. The reason for the different effects of 1Hz rTMS and cTBS when applied to the cerebellum is not clear, as both protocols reduce MEP amplitude when applied to the motor cortex. Moreover, the cerebellar TBS findings were not confirmed by Di Lorenzo et al. [21]. Popa and colleagues [22] studied the effects of 1Hz rTMS, cTBS and iTBS on CBI, rather than motor cortex excitability. Cerebellar cTBS and 1Hz rTMS (but not iTBS) suppressed CBI to the contralateral motor cortex for up to 30 minutes, and they similarly postulated that Purkinje cells were stimulated indirectly. Galea et al. [23] studied the effects of transcranial direct current stimulation (tDCS) on CBI, demonstrating a decrease of CBI with cathodal rDCS and an increase with anodal tDCS.

Cerebellar stimulation has been studied in neurological disorders with some evidence of benefit [24–26], but the mechanisms are incompletely understood, and the optimal protocols uncertain. To investigate this further we used combined TMS and electroencephalography (TMS-EEG) to study the effects of 30Hz TBS on TMS-evoked potentials (TEP). A prominent EEG component following a TMS pulse is the N100 potential—a negative waveform occurring about 100 msec after the magnetic stimulus, thought to reflect intracortical GABA_B_ mediated inhibition [27–29]. It occurs at a similar time point to LICI as demonstrated by paired pulse paradigms, the slope of the N100 correlates with LICI [30] and the N100 amplitude correlates with the cortical silent period (CSP), also thought to reflect inhibitory networks [31]. The N100 amplitude is reduced just prior to movement in a reaction time task [32] and increased by inhibiting a motor response or resisting a pertubation of the wrist [33]. Casula et al. [34] recently demonstrated that 1Hz rTMS to the primary motor cortex, increased the amplitudes of the P60 and N100 components of the TEP and decreased the amplitude of the MEP. However the effects of cerebellar stimulation have not been studied previously.

In the present study, the aim was to investigate the effects of cerebellar TBS on intracortical inhibition, as reflected in the N100 potential, and MEP obtained from the contralateral motor cortex. 30Hz cTBS, iTBS, and sham TBS were applied to the right cerebellar hemisphere in healthy volunteers in three separate experimental sessions, and MEP and TEP were recorded before and after treatment. Two different SI were used for cerebellar stimulation because interim analysis after 8 patients suggested a lack of statistical effect on MEP parameters at the lower SI.

## Methods

### Participants

Sixteen healthy volunteers (nine male and seven female, aged 18–30 years) participated. Thirteen of these were right handed and three left-handed. The exclusion criteria were a medical history of neurological disorder, including epilepsy, and contraindications to transcranial magnetic stimulation, such as cardiac pacemakers, electronic implants, and metal aneurysm clips.

### Electromyography (EMG)

Surface EMG was recorded from the first dorsal interosseous (FDI) muscle of the right hand using 1 cm diameter AgCl disc electrodes with the active electrode placed over the muscle belly, a reference electrode over the metacarpophalangeal joint of the forefinger and a ground electrode on the dorsum of the hand. The electrodes were connected to a Synergy electromyography system (Medelec). Motor evoked potentials (MEP) were recorded with a sampling rate of 48 kHz, high pass filter of 3 Hz, low pass filter of 10 kHz, and sensitivity of 5 mV per division. EMG was recorded for 250 ms after each stimulus.

### Transcranial Magnetic Stimulation (TMS)

A Magstim Rapid^2^ magnetic stimulator and air-cooled 70mm figure of eight coil was used for TMS. A Magstim placebo coil, which mimics the typical “click” of the genuine coil without magnetic stimulation, was used as a control condition. The coil was positioned over the left motor cortex with the handle projecting posteriorly, at a 30–40 degree angle to the mid-sagittal line, to provide a poster-anterior electrical current, approximately perpendicular to the central sulcus [35]. The resting motor threshold (RMT) was defined as the lowest SI required to produce a MEP of at least 50 microvolts (μV) in greater than 50% of trials. The active motor threshold (AMT) was defined as the lowest SI required to produce MEP of at least 200 μV in greater than 50% of trials, while the participant exerted a force of approximately 20 Newtons on a pinch grip dynamometer (B&L pinch gauge). Active and resting MEP were recorded using a SI of 110% of RMT. MEP amplitudes were measured peak to peak, MEP latency was measured from the beginning of the stimulus to the start of the MEP and the CSP was measured as the time from stimulus to the resumption of EMG activity.

### Theta burst stimulation (TBS)

30Hz TBS was applied to the right cerebellum at SI of 80% of AMT for the first eight subjects and SI was increased to 90% for the last eight subjects, after interim analysis of the MEP data suggested lack of effect. The point of stimulation was 3 cm lateral to the midline and 1 cm below the inion [20]. The TMS coil was positioned vertically with the handle placed upwards, as previously shown to optimize inhibition of the contralateral motor cortex [14]. cTBS consisted of 30Hz stimulation, in 3-pulse bursts repeated every 200ms (5 bursts per second) to a total of 600 pulses [7]. For iTBS, two-second trains of bursts were repeated every 10 seconds for a total of 600 pulses. Sham TBS was applied with the placebo coil using the cTBS or iTBS pattern in alternate participants.

### Electroencephalography (EEG)

EEG was recorded using an EasyCap 32 channel electrode cap (Easycap GmbH, Herrsching, Germany) connected to a Synamps RT EEG system (Compumedics Neuroscan, Texas, USA). The following electrodes were positioned on each individual’s head according to the 10–10 electrode placement system: Fp1, Fp2, F7, F3, Fz, F4, F8, FC5, FC1, FC2, FC6, T7, C3, Cz, C4, T8, CP5, CP1, CP2, CP6, P7, P3, Pz, P4, P8, O1, O2. FCz was used as reference electrode. An additional electrode was placed below the right eye, to identify blink artifact. Neuroscan Acquire software was used to record the EEG in DC mode at a sampling rate of 20 kHz, with a high pass filter of 500 Hz. Timing of the magnetic pulses was relayed to the EEG system via a trigger signal from a Digitimer Neurolog System, which simultaneously triggered the Magstim Rapid^2^ stimulator. The Neurolog System was, in turn, controlled by E-Prime software (Psychology Software Tools, Pittsburgh, PA), which produced a signal at pseudorandomised intervals ranging between 3 and 5 seconds. The EEG recordings were down-sampled to 1 kHz offline, and exported to EEGLAB [36], a Matlab-based program (The Mathworks, Inc). Portions of the EEG containing excessive artifact were removed and pre- and post-TBS recordings were combined into a single file for each session. Independent component analysis (ICA) was carried out using the EEGLAB ‘runica’ command. The EEG was epoched from 200 ms prior to the TMS stimulus to 500 ms post-stimulus. The epochs were baseline corrected using the -200 to -50 ms pre-stimulus interval as the baseline. Components containing blink or TMS artifact (approximately 25% of components) were removed. The residual EEG was then processed using principal component analysis and further artifact-containing components were removed. After further baseline correction, the epochs were averaged to obtain the channel event related potentials. A low pass filter of 30Hz (24 dB/Oct) was used for display purposes. The mean TMS-evoked potentials obtained at each electrode before and after each TBS or sham treatment were averaged across subjects and inspected for differences. The N100 amplitude at each electrode was calculated as the mean amplitude of the waveform between 70 and 130 ms after the magnetic stimulus [34].

### Experimental Design

Each participant underwent three sessions carried out at least one week apart. In each session one of the three TBS protocols was applied to the right cerebellar hemisphere (in all participants, irrespective of handedness): 30Hz iTBS, cTBS or sham TBS. The order of the sessions was counterbalanced between participants. The first eight participants received TBS at a stimulus intensity of 80% of AMT (mean = 38.5%, SD = 3.9 of maximum stimulator output) and the second eight participants received TBS at 90% of AMT (mean = 38.9%, SD = 3.9 of maximum stimulator output).

In each session, the electrode cap was placed on the head, and electrodes were applied over the right hand to record MEP. Active and resting motor thresholds were determined, and six to eight resting MEP and a similar number of active MEP were obtained by stimulating the left motor cortex at a stimulus intensity of 110% of RMT. The number of MEP obtained was kept low to minimise carry-over effects on the subsequent TMS-EP. EEG recording then commenced and each participant received 28–32 pulses of TMS to the FDI ‘hotspot’ of the left motor cortex at a stimulus intensity of 90% RMT, at intervals of 3–5 seconds. One of the three TBS protocols was then applied to the right cerebellar hemisphere. After a delay of two minutes a further 28–32 pulses of TMS were applied to the left motor cortex, while EEG was recorded. Finally, motor thresholds and MEP were repeated 5–10 minutes after the end of TBS. White noise was provided through the ear pieces of a stethoscope throughout the experiment, to mask the sound of the magnetic stimulator and reduce contamination of the EEG by auditory evoked potentials.

### Data Analysis

Data were analysed using SPSS version 19 (SPSS Inc., Chicago, USA). The mean thresholds, amplitudes, latencies and cortical silent period, and N100 amplitudes before and after TBS were determined for each session. The Shapiro-Wilk test of normality was applied and variables with non-normal distribution (all except CSP) were first converted to their natural logarithm to reduce the effect of outliers. The N100 amplitudes were converted to positive numerals prior to converting to their natural logarithm. Group analysis was carried out on right handed participants, using a mixed linear model. Left handed participants were excluded due to concerns about the effects of handedness. The dependent variable was the log of the post-treatment value for each parameter and Treatment and SI were fixed factors, with Subject and the log of the pre-treatment value as random factor and covariate respectively. In the case of the N100 amplitude, Electrode was an additional factor (including all cortical electrodes listed above). Post-hoc analyses were carried out using Bonferroni correction. Data was considered significant at *p*<0.05, two tailed.

### Ethics statement

The study was approved by the Lower South Regional Ethics Committee, New Zealand. Written informed consent was obtained from all participants, using a consent form and information sheet approved by the Ethics Committee.

## Results

The procedures were generally well tolerated but one participant who received TBS at the higher SI of 90% AMT withdrew from the iTBS session because of discomfort in the cervical muscles. Two other participants experienced slight discomfort with the higher intensity stimulation, but completed all three sessions. An additional subject experienced transient neck spasm the night after the treatment, and one other subject experienced dizziness the day after their first session, but nevertheless completed all sessions.

After exclusion of left handed participants, 6 participants received TBS at the lower SI of 80% of AMT, 3 female, 3 male, (mean age = 22.33 years, SD = 0.82), and 7 participants received SI of 90% of AMT, 4 female, 3 male, (mean age = 22 years, SD = 2.1).

### TMS evoked potentials

**Fig 2:**
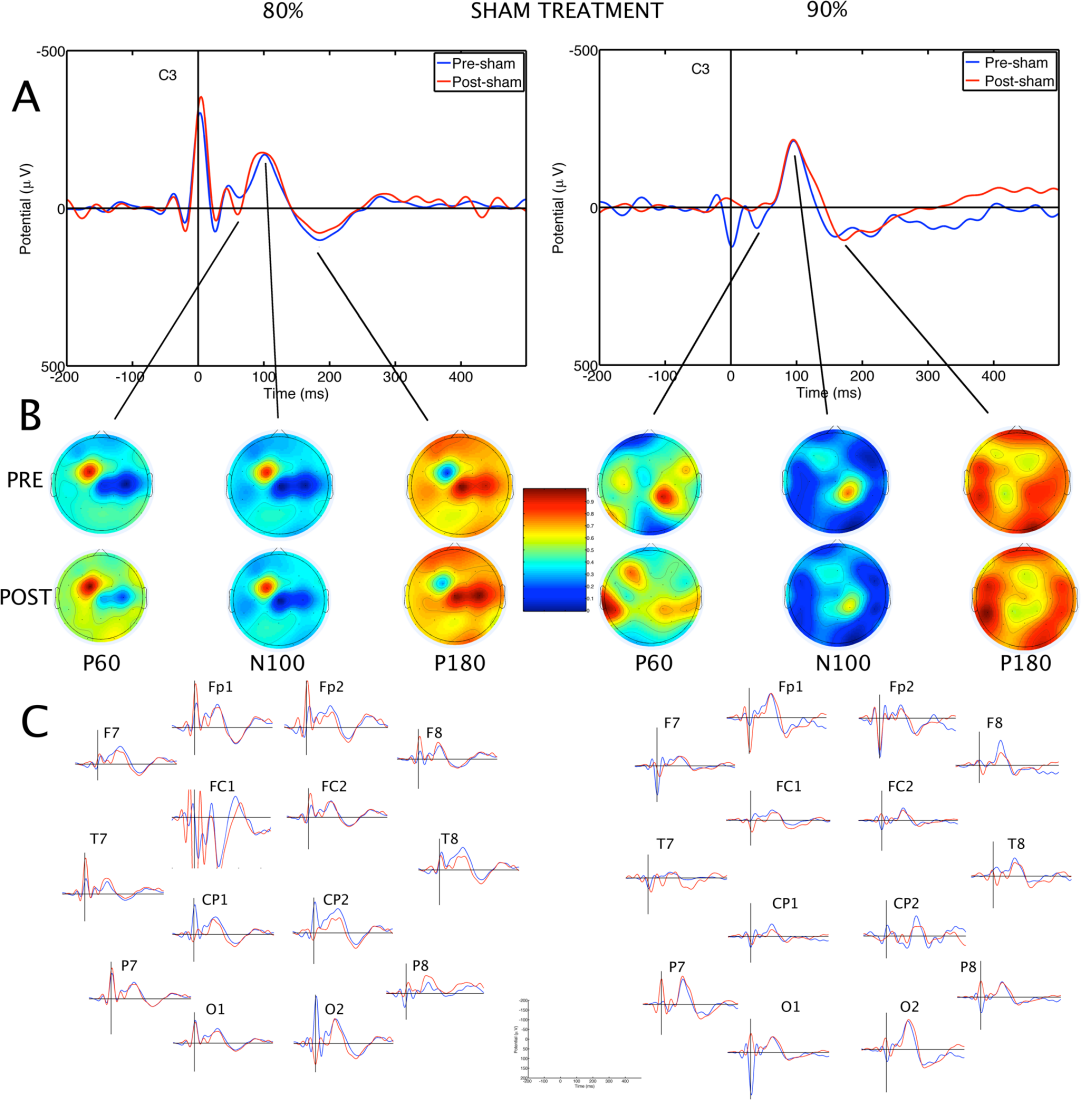
Averaged transcranial magnetic stimulation-evoked potentials before and after sham theta burst stimulation. The effects of sham TBS in the participants who received genuine TBS at 80% of resting motor threshold in other sessions are shown on the left; subjects who received theta burst stimulation at 90% of resting motor threshold are shown on the right. **A.** Grand average pre- and post-stimulation waveforms are shown as recorded at the C3 electrode. B. Topographical distribution of surface voltages for P60, N100 and N180 components of the TEP. Upper maps are pre-treatment, lower maps are post-treatment potentials. C. Grand average pre- and post-treatment potentials at selected electrodes. Blue traces = pre-treatment evoked potentials; red traces = post-treatment evoked potentials; μV = microvolts; msec = milliseconds; TMS = transcranial magnetic stimulation; TBS = theta burst stimulation; RMT = resting motor threshold; TEP = TMS-evoked potential.

**Fig 3:**
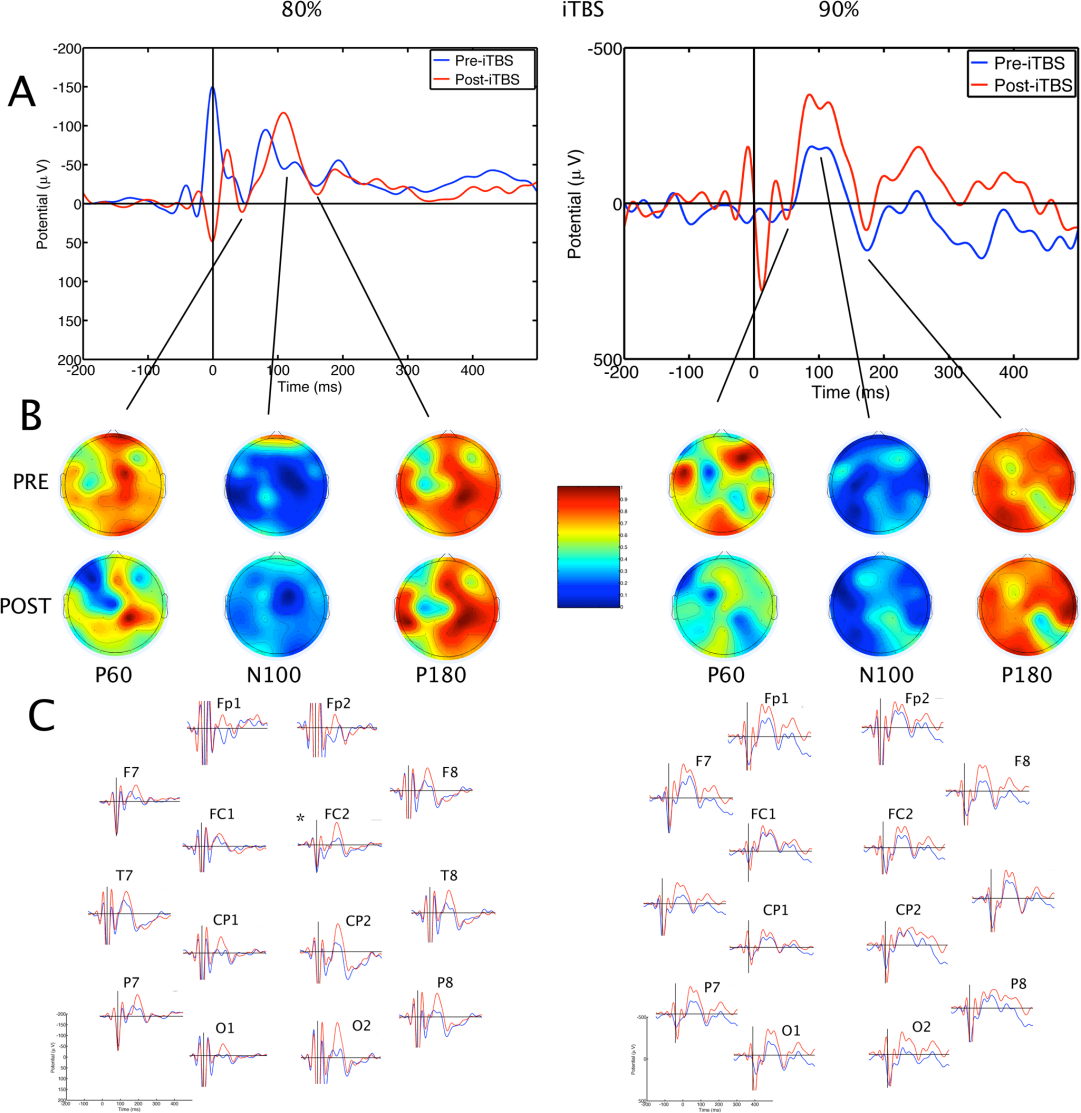
Averaged transcranial magnetic stimulation-evoked potentials before and after intermittent theta burst stimulation. The effects of iTBS at 80% of resting motor threshold are shown on the left; subjects who received intermittent TBS at 90% of resting motor threshold are shown on the right. **A.** Grand average pre- and post-stimulation waveforms are shown as recorded at the C3 electrode. B. Topographical distribution of surface voltages for P60, N100 and N180 components of the TEP. Upper maps are pre-treatment, lower maps are post-treatment potentials. C. Grand average pre- and post-treatment potentials at selected electrodes. Blue traces = pre-treatment evoked potentials; red traces = post-treatment evoked potentials; μV = microvolts; msec = milliseconds; TMS = transcranial magnetic stimulation; TBS = theta burst stimulation; RMT = resting motor threshold; TEP = TMS-evoked potential; iTBS = intermittent theta burst stimulation.

**Fig 4:**
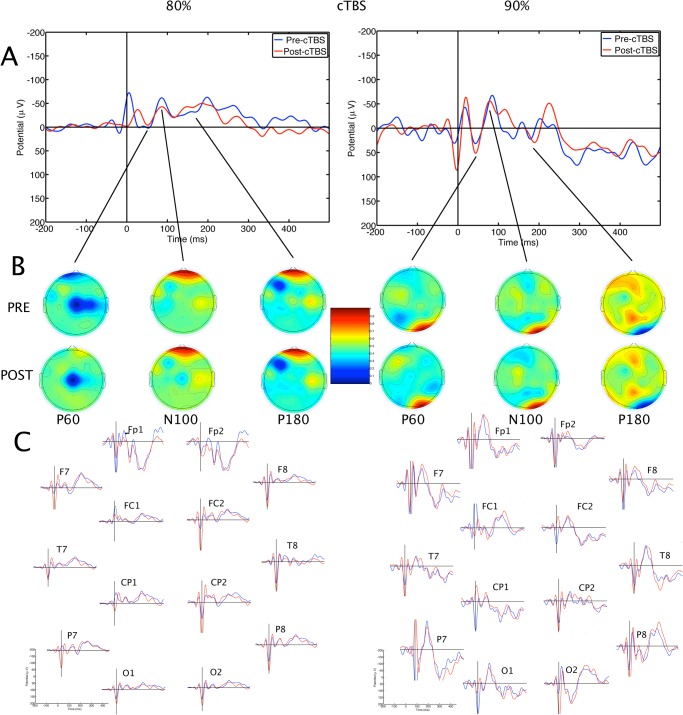
Averaged transcranial magnetic stimulation-evoked potentials before and after continuous theta burst stimulation. The effects of continuous TBS at 80% of resting motor threshold are shown on the left; the effects of continuous theta burst stimulation at 90% of resting motor threshold are shown on the right. **A.** Grand average pre- and post-stimulation waveforms are shown as recorded at the C3 electrode. B. Topographical distribution of surface voltages for P60, N100 and N180 components of the TEP. Upper maps are pre-treatment, lower maps are post-treatment potentials. C. Grand average pre- and post-treatment potentials at selected electrodes. Blue traces = pre-treatment evoked potentials; red traces = post-treatment evoked potentials; μV = microvolts; msec = milliseconds; TMS = transcranial magnetic stimulation; TBS = theta burst stimulation; RMT = resting motor threshold; TEP = TMS-evoked potential; cTBS = continuous theta burst stimulation.

**Fig 5:**
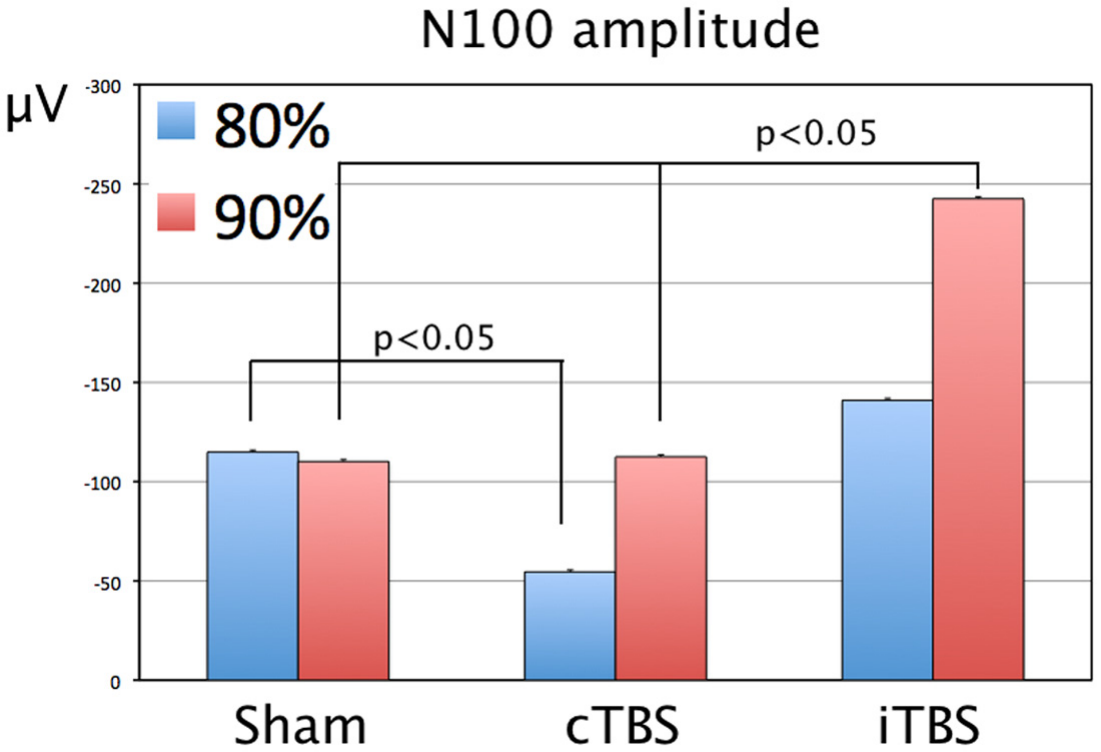
Effects of cerebellar cTBS, iTBS and Sham TMS on the N100 potential in right handed participants. The post-treatment marginal means for the interaction Treatment x Stimulus Intensity are shown for the N100 amplitude. p values are shown for the post-hoc comparisons, using Bonferroni correction. Error bars show standard errors. Sham = sham treatment with placebo coil; iTBS = intermittent theta burst stimulation; cTBS = continuous theta burst stimulation; μV = microvolts.

The TMS-evoked potentials consisted of a series of positive and negative waveforms including the P60, N100 and N180 potentials (Figs 2, 3 and 4). Some of the earlier waveforms were contaminated by residual TMS artefact, despite artefact removal. Mixed ANOVA with factors Treatment x SI x Electrodes, with pre-treatment N100 amplitude as a covariate revealed a significant effect of Treatment on N100 amplitude, F(2,494) = 41.06, p < 0.001. Post-hoc analysis showed that values were significantly higher (more negative) after iTBS than after Sham or cTBS, p < 0.05. There was an effect of SI on N100 amplitude, with higher amplitudes after 90% than 80% SI, F(1,576) = 19.46, p<0.001. There was a significant Treatment x SI interaction, F(2,494) = 7.88, p<0.001. Post-hoc comparisons showed that the mean N100 amplitude after iTBS at 90% SI was higher than sham or cTBS at 90% SI (Fig 5). In contrast, cTBS at 80% resulted in lower mean N100 amplitude than sham treatment or iTBS. There was no significant difference between 80% iTBS and sham. There was no effect of Electrode, and the effects appeared to be generalised, rather than localized to a few electrodes (Fig 3B and 3C). The mean N100 amplitudes for each participant are contained in the supplementary file S1 N100 Data.

### Motor evoked potentials

**Fig 6:**
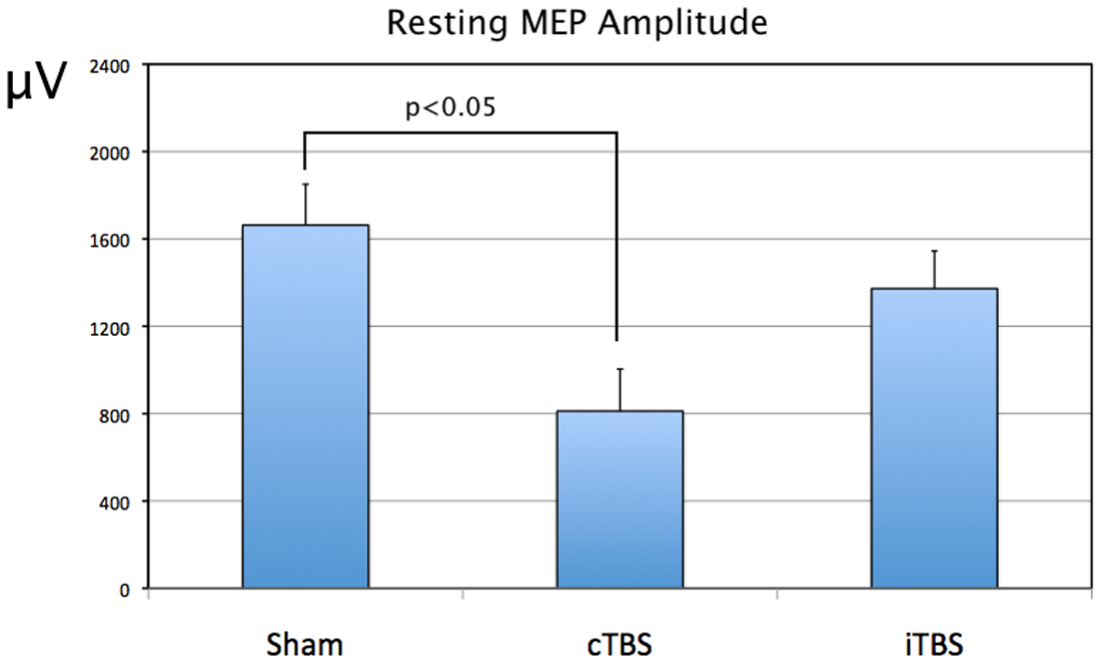
Effects of cerebellar cTBS, iTBS and Sham TMS on the resting motor evoked potential in right handed participants. The post-treatment marginal means for the interaction Treatment x Stimulus Intensity are shown for the resting MEP amplitude. p values are shown for the post-hoc comparisons, using Bonferroni correction. Error bars show standard errors. Sham = sham treatment with placebo coil; iTBS = intermittent theta burst stimulation; cTBS = continuous theta burst stimulation; μV = microvolts.

There was an effect of Treatment on resting MEP amplitude, F(2,20) = 3.63, p = 0.045, where post-hoc analysis demonstrated a significantly lower amplitude following cTBS as compared to sham. (Fig 6). There were no significant effects or interactions for active MEP amplitude, CSP, thresholds, or latencies at the p<0.05 threshold. The mean variables for each participant are contained in the supplementary file S1 MEP Data.

## Discussion

In this study, stimulation of the cerebellum with 30Hz iTBS diffusely increased the amplitude of the N100 waveform of the TEP compared to sham treatment, with no significant change in MEP amplitude. The effect on the N100 was largely due to iTBS at the higher SI of 90% of AMT. In contrast, cTBS at 80% (but not 90%) AMT decreased N100 amplitude and cTBS reduced resting MEP amplitude when both SI were combined. The dissociation of MEP and N100 effects highlights the fact that MEP amplitudes are dependent on a combination of cortical and spinal mechanisms and represent, at cortical level, a balance between inhibitory and facilitatory intracortical networks [37,38]. The findings suggest that the effects of cerebellar cTBS are most likely mediated via alterations in facilitatory networks, while iTBS exerts an effect on inhibitory networks (as reflected in the N100 wave amplitude). Casula et al. [34] found no correlation between changes in MEP and the N100 in a study where neuromodulation was achieved with 1 Hz rTMS applied directly to the motor cortex.

Koch and colleagues [20] reported that 50Hz cerebellar cTBS decreased MEP amplitude. In their study, iTBS at 80% of AMT increased resting MEP amplitude, which we were unable to demonstrate. Although they did not examine the N100, the effects on LICI are in contrast to our finding of increased N100 amplitude with iTBS, considering that both reflect intracortical inhibition. Di Lorenzo et al. [21] found no changes in MEP amplitude, but did not study TEP. Those studies differ from the present study by the use of 50Hz rather than 30Hz TBS. The effects of 30Hz TBS applied to the motor cortex have, however, been reported to be similar to those obtained with 50Hz TBS: reduced amplitude following cTBS and increased amplitude following iTBS [8,9,39].

The axons of the Purkinje cells constitute the sole output to the deep cerebellar nuclei [22], and receive inhibitory input from the (more superficial) stellate and basket cells of the molecular (superficial) cerebellar cortical layer. If TBS has a direct effect on Purkinje cells, one could postulate that (excitatory) iTBS would increase the activity of Purkinje cells, and the result would be increased inhibition of the (excitatory) dentate-thalamo-cortical pathway resulting in decreased MEP amplitudes obtained from the contralateral motor cortex. Inhibitory cTBS would have the opposite effect: disinhibition of the pathway with increased MEP amplitudes. This was found neither in this study, nor by Koch et al. [20]. On the other hand, if TBS acts directly on cells of the superficial cortical layer which have inhibitory synapses on Purkinje cells, the effects along the pathway would be reversed, in keeping with the findings of Koch et al. [20]. We suggest that the effects of cerebellar TBS may be highly dependent on SI and coil type. Lower SI is less likely to modulate Purkinje cells directly, and more likely to exert its effects via cells in the molecular layer which themselves inhibit Purkinje cell activity. This may explain why lower stimulus intensity of cTBS resulted in decreased resting MEP amplitude. Individual variation in the depth of the cerebellar cortex will also affect which cells are stimulated. Some researchers have attempted to adjust for the depth of the cerebellum, by correcting the SI on the basis of cerebellar imaging [22]. Others have recommended the use of a double-cone coil [40]. Thus SI, coil type and local anatomy may be critical in cerebellar stimulation, and possibly explain variation between studies and between individual subjects. Hamada et al [41] have shown considerable variation between individuals in response to theta burst stimulation of the motor cortex, possibly due to differences in recruitment of cortical neurones, and similar mechanisms may apply in the cerebellum.

Cortical excitability is important in neurological disease. Excitation is mainly mediated by glutamate, acting at N-methyl-d-aspartate (NMDA) and non-NMDA receptors, and inhibition is largely mediated by GABA, acting on GABA_A_ or GABA_B_ receptors [3]. The N100 potential is thought to reflect intracortical inhibition [27,28]. Premoli et al. [42] studied the effects of GABAergic drugs on TMS-EEG, showing that the N45 and N100 potentials can be linked to GABA_A_ and GABA_B_ neurotransmission respectively. Reduced N100 amplitude has been demonstrated in attention deficit disorder [28, 43]; alcohol ingestion [44]; Unverricht-Lundborg type progressive myoclonic epilepsy [45]; and mild cognitive impairment [46].

Non-invasive cerebellar stimulation has been trialled in a few neurological diseases. Two weeks of cTBS resulted in clinical improvement in dystonia, a condition associated with impaired GABA inhibition [25,47]. Patients with Parkinson Disease (PD) appear to have dysfunctional CBI [48,49] and levodopa-induced dyskinesia in Parkinson disease (PD) may benefit from cerebellar cTBS [50]. In epilepsy, Brighina et al. [51] produced a significant decrease in seizure frequency in drug-resistant patients, using 20 daily treatments with 5Hz cerebellar rTMS.

The rational use of therapeutic neuromodulation aims to target either inhibitory or facilitatory networks and it is important to better understand which target is appropriate for a particular disease. Paired pulse TMS provides measures of intracortical facilitation and inhibition, useful in studying cortical excitability in disease, while TMS-EEG provides a powerful additional measure, applicable in cortical areas beyond the motor cortex. Further studies on neurological disorders should aim to elucidate whether the N100 and other components of the TEP can provide biological markers to assist in developing neuromodulatory treatments. Further studies on healthy volunteers are required to elucidate the effects of rTMS protocols on those markers. The findings of the present study suggest that cerebellar iTBS, rather than cTBS may be an effective way to increase intracortical inhibition, and may provide an alternative to inhibitory rTMS protocols applied to the cerebral cortex.

A limitation of the present study is that we did not study paired pulse measures of cortical excitability. We were therefore unable to relate the N100 findings directly to SICI and LICI. MEP were not obtained concurrently with the TEP: TEP were studied two minutes post-stimulation, while MEP were studied at 5–10 minutes post stimulation. We did not use a correction factor to determine SI, to compensate for the depth of the cerebellar cortex in each subject. Importantly, we did not counterbalance the two SI that were used: the first 8 participants received TBS at 80% and the rest received 90% of AMT. Comparisons between TBS at 80 and 90% were therefore unreliable, as there may have been a period effect. Another concern in studies of cerebellar TMS is that the findings could result from stimulation of the cervical nerve roots, which are close to the site of stimulation, as evidenced by occasional neck muscle twitching seen during stimulation. A previous study suggested that cerebellar TMS has both central and peripheral actions [17], but other studies concluded that it was unlikely that results of cerebellar stimulation were due to nerve root stimulation [22]. We confined our analysis to right handed participants, out of concern that handedness may be important in cerebello-cortical effects. There were insufficient left handed participants to study this specifically.

Despite these limitations, we have demonstrated that cerebellar iTBS at the higher SI increased the amplitude of the N100 potential, thought to reflect GABA_B_ intracortical networks, while cTBS at lower SI reduced it. It is likely that these effects are exerted via the dentate-thalamo-cortical pathway. The findings suggest that cerebellar TBS may be a useful therapy for neurological disorders. Specifically, cerebellar iTBS at the higher SI would be a logical paradigm for disorders characterised by impaired GABA_B_ inhibitory mechanisms, whereas low intensity cTBS may be more appropriate where facilitatory networks are overactive.

## Supporting Information

S1 MEP Data Contains MEP parameters for each experimental session. (XLSX)

S1 N100 Data Contains N100 amplitudes for each experimental session. (XLSX)
